# Defect Detection of Industry Wood Veneer Based on NAS and Multi-Channel Mask R-CNN

**DOI:** 10.3390/s20164398

**Published:** 2020-08-06

**Authors:** Jiahao Shi, Zhenye Li, Tingting Zhu, Dongyi Wang, Chao Ni

**Affiliations:** 1 College of Mechanical and Electronic Engineering, Nanjing Forestry University, Nanjing 210037, China; jiahaoshixhl@163.com (J.S.); zhenye@njfu.edu.cn (Z.L.); tingting_zhu2018@163.com (T.Z.); 2 Bio-Imaging and Machine Vision Lab, Fischell Department of Bioengineering, University of Maryland, College Park, MD 20740, USA; dywang@umd.edu

**Keywords:** wood veneer defect detection, online detection, Neural Architecture Search (NAS) technology, multiple channel mask R-CNN

## Abstract

Wood veneer defect detection plays a vital role in the wood veneer production industry. Studies on wood veneer defect detection usually focused on detection accuracy for industrial applications but ignored algorithm execution speed; thus, their methods do not meet the required speed of online detection. In this paper, a new detection method is proposed that achieves high accuracy and a suitable speed for online production. Firstly, 2838 wood veneer images were collected using data collection equipment developed in the laboratory and labeled by experienced workers from a wood company. Then, an integrated model, glance multiple channel mask region convolution neural network (R-CNN), was constructed to detect wood veneer defects, which included a glance network and a multiple channel mask R-CNN. Neural network architect search technology was used to automatically construct the glance network with the lowest number of floating-point operations to pick out potential defect images out of numerous original wood veneer images. A genetic algorithm was used to merge the intermediate features extracted by the glance network. Multi-Channel Mask R-CNN was then used to classify and locate the defects. The experimental results show that the proposed method achieves a 98.70% overall classification accuracy and a 95.31% mean average precision, and only 2.5 s was needed to detect a batch of 50 standard images and 50 defective images. Compared with other wood veneer defect detection methods, the proposed method is more accurate and faster.

## 1. Introduction

Wood is an essential natural resource, but defects on wood products can seriously affect the commercial value. Due to the low quality of raw materials and inappropriate manufacturing processes, there are various kinds of defects on wood veneers, such as live knots, dead knots, and cracks. These defects diminish the utilization of raw wood materials in some developing countries. Visual quality inspections are still mainly performed by trained workers in the wood processing industry [1,2].

A fast wood defect detection method is necessary for modern wood veneer processing industries to improve their wood use rate and increase their revenue. Currently, many kinds of technologies have been used to detect defects on wood veneers, including air-coupled ultrasonic technology [3], stress wave technology [4], 3D laser technology [5], computed tomography [6], and computer vision technology [7]. Air-coupled ultrasonic technology is a contactless ultrasonic measurement method that detects defects inside the wood veneer based on defects’ densities variations inside the wood. However, ultrasonic technology is susceptible to the external environment and lacks stability, which leads to unacceptable detection efficiency for industrial requirements [8]. Stress wave technology can detect the size and shape of internal defects based on segmented propagation rays of stress waves. However, the acquisition of stress wave signals requires the sensors to be firmly attached to the surface of the wood and the distance between sensors must be fixed, which restricts the efficiency of defect detection and makes this method not flexible enough for industry usage [9]. 3D laser technology can be used to accurately measure the shape of wood surfaces and identify defects with 3D scanner technology in terms of area and volume [10]. However, some defects cannot be detected accurately by the 3D laser scanner when their shapes are very small. Computed tomography-based X-ray is used to separate out knots of wood based on density and moisture, so that the accuracy is most dependent on the moisture, which would result in the thick wood separating into knots [6]. Computer vision technology is an efficient method to deal with images and videos and has been used to detect various defects [11,12]. For example, Boardman et al. [13] used colorimetric techniques to detect defects in black walnut veneer. Momin et al. [14] used an Hue-Saturation-Intensity (HIS) color model coupled with median blurring, morphological operators, and watershed transformation to detect dockage fractions. However, the HIS color model cannot be extended to detect all wood defects due to the variety of defects. Dawood et al. [15] proposed an integrated model supported by regression analysis to predict spalling depth. However, the performance of the integrated model was sensitive to input data, and thus its robustness was poor.

With the development of computer hardware, especially high-performance Graphics Processing Units (GPUs), the combination of computer vision technology and deep learning technology has the advantages of fast speed and low cost, and has been widely used in defect detection [16]. Park et al. [17] proposed a method for automatic visual inspection of defects on the surface of wood products based on a convolution neural network (CNN), which was an effective algorithm for wood defect detection, but was not put into actual application. Jung et al. [18] employed three different architectures of CNN to classify regular wood and four types of defect images. Comparing the performance of the three CNN models, the deep CNN achieved a high classification accuracy of 99.8% for defect detection, but its running speed was slow because the network went deeper. He et al. [19] used a mixed fully convolutional neural network (Mix-FCN) to locate and classify wood defects with the wood surface images automatically. However, the Mix-FCN used all of the computing resources for all of the wood regardless of the majority of wood images being free of defects, which was inefficient time-wise. Urbonas et al. [20] applied a pre-trained ResNet152 neural network model combined with faster R-CNN to find wood panel surface defects and achieved an accuracy of 96.1%. However, this method only output a box containing the wood defects instead of the real defect shapes, which affected the precision of the defect incision.

Mask R-CNN was developed as an improved version of faster R-CNN, which can detect defects and output the defect shapes; it has been widely used in the field of target detection, and achieves excellent performance in many applications. In mask R-CNN, the image is processed by a series of convolution layers and passed through three different branches of networks [21]. However, few studies applied it to wood defect detection. Huang et al. [22] reported a method of assembly inspection based on the mask R-CNN model for identifying and drawing the defects from each part of the image and achieved a classification accuracy of over 86.5%. Hu et al. [23] used mask R-CNN coupled with a generative adversarial network (GAN) to identify and classify veneer defects, and the accuracy reached 98.4%. Although the detection accuracy of mask R-CNN is high, the slow detection speed is its unignorable disadvantage [24]. Therefore, it is necessary to develop a method considering the accuracy and running speed for wood defect detection for industrial production.

In this study, a glance network was added before the mask R-CNN to reduce the running time of the mask R-CNN. The glance network was used to filter out all defective pictures and mistake as few regular pictures for defective pictures as possible. Simultaneously, the glance network should use as few computing resources as possible during classification. Although the goals of the glance network were clear, it was hard to find the most suitable architecture and parameters to satisfy them. Hence, neural network architecture search (NAS) technology was used to construct the structure and determine the parameters of the glance network.

NAS is an emerging technology that constructs different kinds of structures based on intelligent algorithms [25,26,27]. At present, most neural network architectures are manually developed and carefully designed by human experts, which is a time-consuming and error-prone process. Therefore, NAS was developed to build new structures for a more accurate target [28,29,30]. However, NAS is only used for improving the accuracy of the neural network while ignoring the real-time performance of the constructed architecture, which makes it hard to adapt to industrial production.

In this research, an improved method that combined NAS technology and mask R-CNN was applied to detect defects on wood veneer surfaces and output the shape and type of defects. The proposed algorithm integrates an optimized multi-channel mask R-CNN and a glance network based on NAS to obtain a fast scan of the input image for distinguishing defective wood veneer images from all wood veneer images. In contrast to standard NAS technology, in the glance network, the speed of the neural network is considered using the value of floating point operations (FLOP). Then, the glance network established by the NAS extracts and outputs the first impression features of defect images to the following multi-channel mask R-CNN for further detection. To have the best combination of features input into the multi-channel mask R-CNN, a genetic algorithm is used to optimize the feature selection for achieving better detection results. The proposed integrated model can ensure detection accuracy and better real-time performance compared to the traditional mask R-CNN, which lacks a glance network and a multi-channel structure.

Specifically, the integrated model is used as the final classifier to identify the defects on the wood veneer surface, and the specific contributions of this work are as follows:

- A glance network is developed for quickly scanning the image to determine whether the image is suitable for further detection, which significantly reduces detection time for industrial usage. Additionally, a new fit value function utilizing the FLOP of the network is constructed for the first time to improve the real-time performance of the glance network.
- A genetic algorithm is used to determine the feature selection of the multi-channel mask R-CNN input channels in order to achieve higher detection accuracy.

## 2. Materials and Methods

### 2.1. Materials and Data Collection

**Figure 1:**
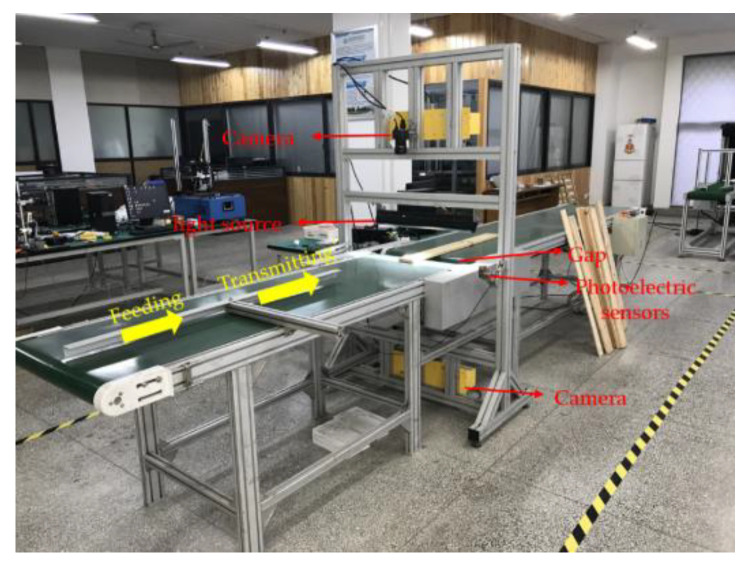
Acquisition equipment used to collect wood veneer images.

Wood veneer images were collected using acquisition equipment as shown in Figure 1. The acquisition equipment included two cameras (Chroma + Scan3350), a conveyor belt, a light source, and a photoelectric sensor. The two cameras were on the top and bottom of the gap in the belt, and they normally work for recording images with 8-bit depth between the temperatures of 0 and 65 °C. The width of the conveyor belt was 0.6 m and the length was 4.5 m, and its moving speed was 1.5 m/s. The height of the camera above the conveyor belt was 0.56 m. The height of the light source above the conveyor belt was 0.2 m.

**Figure 2:**
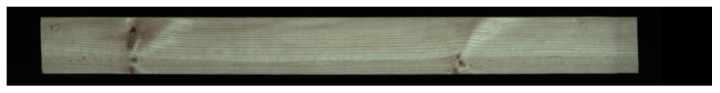
One-side photo of a wood veneer collected in a single run by the data acquisition equipment.

The photoelectric sensor is ES12-D15NK produced by LanHon in Shanghai, China, and its detection distance is up to 15 cm. It was attached to the gap of the belt and produced a pulse to the acquisition board on the computer. When the wood veneer was detected to reach the gap, the two cameras started scanning the wood veneer. The scanning process stopped when the wood veneer leaves the gap. In this way, the two sides of the wood veneer were obtained in one scanning process. The experimental wood discussed in this paper was pieces of Chinese fir with dimensions 1000 × 100 × 10 mm, which were picked out by trained workers from a wood processing company (Jiangsu Jiangjia Machinery Co., Ltd., Yancheng, China). A scanning photo of a wood veneer including the background had the dimensions 18,000 × 2048 × 3 (width × height × channels), as shown in Figure 2.

**Figure 3:**
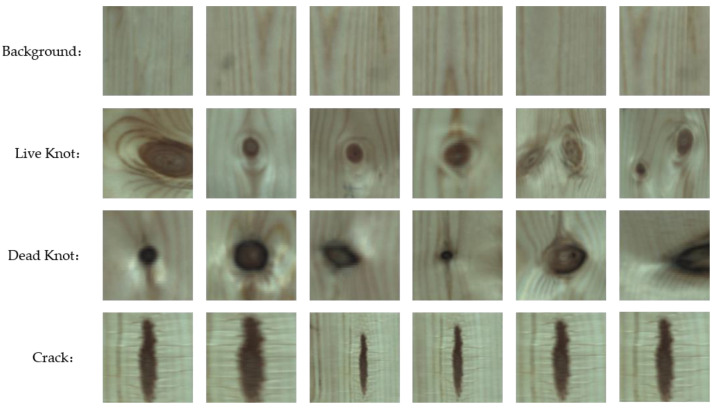
Regular wood pieces as background and three common wood defects taken from the dataset.

To construct an appropriate dataset for experiments, the black background from the raw pictures was first removed. Then, the pictures were cut into 200 × 200 pixel pieces. After that, the pictures were processed by rotation, magnification, and horizontal and vertical mirroring to expand the quantity of the wood veneer image set. Finally, 2838 wood veneer pictures were obtained, of which 612 were regular pictures (background), and the remaining 2226 pictures each contained one or more defects, including 846 live knot pictures, 760 dead knot pictures, and 620 crack pictures, as shown in Figure 3. Among these defective images, the area of defects ranges from 12.086 to 313.238 mm^2^.

**Figure 4:**
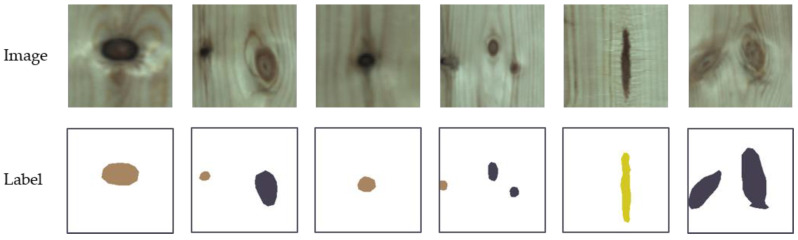
Defect images and corresponding label areas in the dataset. In label images after visualization, white represents the background, brown represents dead knots, dark blue represents live knots, and dark yellow represents cracks.

Trained workers used LabelMe software to label wood defects. LabelMe is a graphical image annotation tool written in Python that uses Qt as its graphical interface. LabelMe can carry out polygon annotation and output a COCO-format dataset, such as segmentation, which is helpful for picking wood defect masks from the images. The label results of defect images are shown in Figure 4.

### 2.2. Method

**Figure 5:**
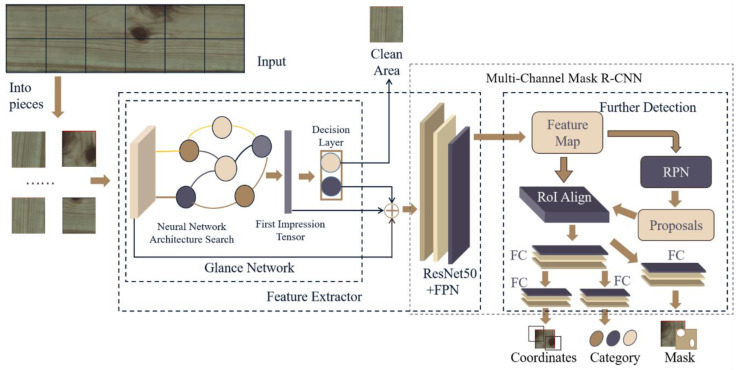
Structure chart of the target detection algorithm for wood defects detection. FPN: feature pyramid network; FC: fully connected network; RPN: region proposal network.

The process of the proposed target detection algorithm is shown in Figure 5, and it includes three main parts: (1) Preliminary classification: Each input picture is classified by a glance network to determine whether the picture is a defective picture. The pictures with defects will be output to the following network. (2) Feature extractor: The first impression tensors of a picture are extracted by the glance network and sent with a defect picture input to the ResNet50 network and the feature pyramid networks (FPN) for extracting further features. (3) Defects detection: The feature maps extracted above are input into the region proposal network (RPN) and region of interest (RoI) Align, separately. The RPN marks rectangular candidate regions containing defects in the image. RoI Align is used to derive higher level feature maps from the RoI by combining the feature maps and the RPN marked region. Then, these high-level feature maps are input to several fully connected (FC) layers to obtain the type, the coordinates, and the mask of defects.

#### 2.2.1. Glance Network Based on NAS for Speed Improvement

**Figure 6:**
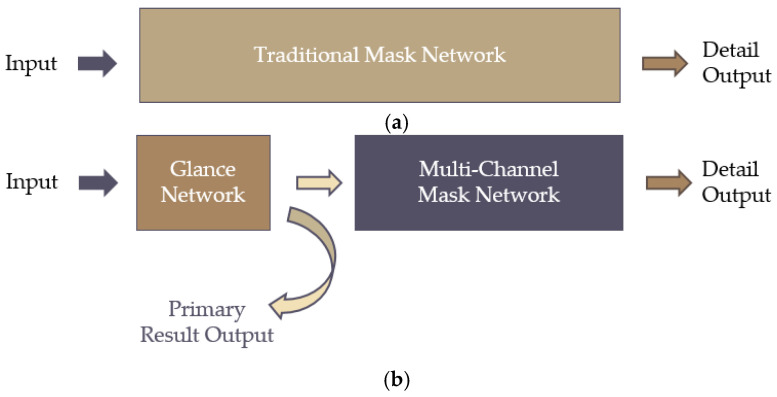
Structure difference between traditional mask network and the improved model. (**a**) Traditional mask network model, (**b**) Improved mask network model with glance network.

In the real industrial wood detecting process, wood defects only account for a small part of the whole wood, as shown in Figure 2. Thus, computing resources would be wasted in scanning and processing the whole wood image through the whole mask detection model for defects, as shown in Figure 6a. In this study, a relatively simple glance network was used to distinguish defective wood images from massive regular wood images before the mask detection network, as shown in Figure 6b. In this way, only defective images were input through the whole network, which effectively saved computing resources and accelerated the whole detection process. Specifically, the glance network has a more straightforward structure than a mask network model, and thus its processing speed is faster than the traditional mask network. During the preliminary classification, the requirements for classification are lower than mask defects, so that the glance network is more suitable than a mask network model for classifying regular images and detective images. The features of defective images are extracted by the glance network and called the first impression. The first impression and defective images are transferred to a multi-channel mask network.

**Figure 7:**
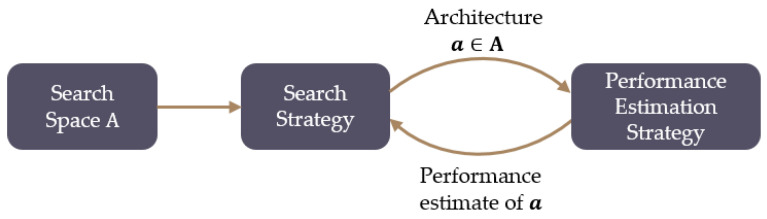
Flow path of neural architecture search technology.

To achieve accurate detection performance, NAS technology was used to design an appropriate structure for the glance network. Deep learning was successfully applied in perceptual tasks with its advantage of automatic feature extraction from data rather than by manual design [31]. NAS is a method of automated architecture engineering, and it is used to replace the traditional human hand-built neural network architecture. The superiority of NAS has been proven by many experimental results in object detection or semantic segmentation [32,33,34]. The NAS method is divided into three parts: a search space, a search strategy, and a performance evaluation strategy, as shown in Figure 7. A search strategy selects an architecture from the predefined search space **A**. The architecture is passed to a performance estimation strategy, which returns the estimated performance of ***a*** to the search strategy.

In this study, a coding method was developed to determine the search space of the glance network architecture. Here, the function of the glance network was to analyze the input picture as fast as possible based on a convolution network, which has been successful in many applications [35,36,37,38,39,40]. The search space of the glance network architecture was divided into eight identical code blocks, and each block contained a series of predefined building parameters listed in Table 1. Since the glance network was based on a convolutional neural network, these parameters included the type of layer, whether to activate, the basic parameters of the convolutional layer, and the max-pooling layer. The parameters of each network structure were represented with fixed-length binary strings to construct the search space of the glance network.

After determining the search space for the network architecture, the network was limited to a fixed depth, but there were still many candidate networks. Therefore, a suitable search strategy was necessary to effectively search for the desired glance network in the vast search space. The search strategy of the glance network structure not only has to find the architecture with excellent performance but should also avoid premature convergence to a sub-optimal architecture area. Here, the search strategy is transformed as a two-level optimization problem [41]:

(1) $$\begin{array}{l} \underset{N_{\alpha }^{w}\in N_{A}}{\min}C_{\mathrm{valid}}(N_{\alpha }^{w^{*}})\\ s.t.w^{*}=\underset{w}{\arg\min}L_{\mathrm{train}}(N_{\alpha }^{w}) \end{array}$$

where $N_{\alpha }^{w}$ is the network with architecture α**,** $N_{A}$ is the collection of all the possible network architectures, $C_{\mathrm{valid}}$ is the evaluation criterion on the validation dataset, $w^{*}$ represents the best weight value for the network, and $L_{\mathrm{train}}$ is the loss function on the training dataset.

**Figure 8:**
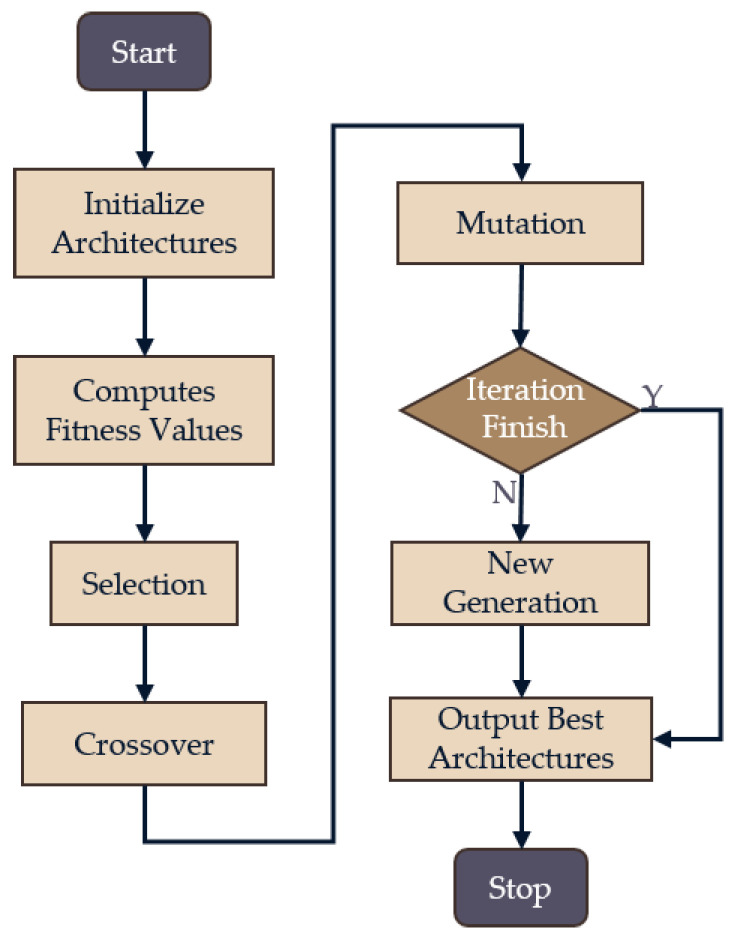
Flow chart for neural network architecture search (NAS) with genetic algorithm.

To solve the two-level optimization problem, a genetic algorithm was used for the optimization of $C_{\mathrm{valid}}$ and Stochastic Gradient Descent–Momentum (SGD-M) was used for the optimization of $w^{*}$. The genetic algorithm has been widely used for NAS, and it has high robustness in many applications [42,43,44,45]. By using the genetic algorithm, the original traversing each structure to select the best problem was transformed into optimizing in a large search space, which efficiently traversed the space. The main optimization steps of the genetic algorithm were initialization, selection, mutation, crossover, and individual evaluation, as shown in Figure 8.

For industrial applications, both the accuracy and the speed of the proposed model were considered by designing a new kind of fitness value (F), which is represented as:

(2) $$F=2-F_{s}-F_{\mathrm{acc}}$$

where $F_{s}$ stands for the fitness value of speed and $F_{\mathrm{acc}}$ stands for the average accuracy of the model.

(3) $$\left\{ \begin{array}{l} F_{s}=e^{-(\alpha f+\beta )}\\ \alpha =\frac{\ln2}{f_{\max}-f_{\min}}\\ \beta =-\frac{\ln2f_{\min}}{f_{\max}-f_{\min}} \end{array}\right.$$

where f, $f_{\max}$, and $f_{\min}$ represent the number of floating-point operations (FLOPs) of a model and the maximum and minimum values of all possible neural architectures, respectively. α and β are the parameters for the scaling of FLOPs. Here, the number of FLOPs was used as a metric for the speed of the neural network, which was an indirect method to measure the complexity and the running speed of a model [41]. The FLOPs’ values of common layers are defined as [46]:

(4) $$\left\{ \begin{array}{l} FLOPs_{conv}=2HW(C_{in}K^{2}+1)C_{out}\\ FLOPs_{\max p}=H_{out}W_{out}C_{\mathrm{out}}K^{2}\\ H_{out}=\frac{H-K}{s}+1,W_{out}=\frac{W-K}{s}+1 \end{array}\right.$$

where $FLOPs_{conv}$ is the FLOPs of a convolutional layer and $FLOPs_{\max p}$ is the FLOPs of a max-pooling layer;H,W, and $C_{in}$ are the height, width, and the number of channels of a feature map, respectively; K is the kernel size of the layers; and $C_{\mathrm{out}}$ is the number of output channels.

Assume the maximum number of layers in the architecture space is n, and every convolution layer has the same padding. Then, we can obtain the expression:

(5) $$\left\{ \begin{array}{l} f_{\max}=nHW(2k^{2}c^{2}+k^{2}+2c)+f_{\min}\\ f_{\min}=2HW(dk^{2}+1)+HW(k^{2}+4)-4 \end{array}\right.$$

where H,W and d are the height, width, and the depth of the input picture, respectively; k is the kernel size of the layers; and c is the input channel of the feature. The other factor of the fitness value is the average accuracy of the model and is defined as:

(6) $$F_{\mathrm{acc}}=\sum \frac{C_{i}{}^{T}}{C_{i}}/C_{n}$$

where $C_{i}^{T}$ is the correct classification number of the class i, $C_{i}$ is the total number of the class i, and $C_{n}$ is the number of classes.

**Figure 9:**
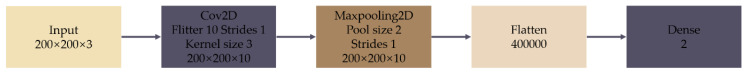
Structure of glance network constructed by NAS.

After 66 h of neural architecture searching, the structure of the glance network was determined as shown in Figure 9. The structure consisted of a convolutional layer with 280 trainable parameters, a max-pooling layer, a flatten layer, and a dense layer with 800,002 trainable parameters.

#### 2.2.2. Feature Selection for Accuracy Improvement

During the detection of the glance network, some features of images are already extracted from the images to construct the result. To apply these features to achieve higher accuracy for the following mask network, the previous features skipped by the glance network are merged with the input images. This method has little effect on the detection speed of mask R-CNN, but it effectively improves the detection accuracy, as shown in the results listed in Table 2.

The convolution layer can extract different features based on different kinds of convolution kernels, and the output of a convolution layer can be expressed as:

(7) $$z(u,v)=\sum_{i=-\infty }^{\infty }\sum_{j=-\infty }^{\infty }x_{i,j}\cdot p_{u-i,v-j}$$

where p represents different kinds of kernels at different pixels, z(u,v) is the output of one channel of the convolutional layer, and $x_{i,j}$ represents the raw input images.

Assuming that the latest convolution layer of glanced networks has n channels, the channels can be regarded as a list of features. Since different channels have different methods of feature extraction, some features are critical for the following defect detection while the others are not. A genetic algorithm (GA) is used to search for the best combination of the first impression features extracted from the glance network. The search space of the channels is encoded into an array with the shape of n × 1. Each value of the array is an enumerated value, either 0 or 1, which represent “drop out” and “chosen”, respectively. Since the following mask network cares only about the accuracy of the detection, the average accuracy of the model is used as the metric of the GA.

#### 2.2.3. Multi-Channel Mask R-CNN

The NAS method is used to search for a high-performance glance network as a front-end network, and then the defect images and first impressions are input into the mask R-CNN network to determine the type and precise location of wood defects in Figure 5. The mask R-CNN network structure is an end-to-end convolutional neural network proposed by the Facebook artificial intelligence research group, and it has an excellent detection effect in achieving target instance segmentation. It can accurately detect and mark the targets, and generate a high-quality segmentation mask for each detected target.

Multi-scale detection is becoming increasingly critical in target detection, especially for small targets, and feature pyramid network (FPN) is a well-designed multi-scale detection method. Therefore, mask R-CNN uses ResNet50 and FPN for further feature extraction following glance network classification. FPN uses feature maps in the form of a pyramid, integrating these feature maps efficiently through bottom-up, top-down, and lateral connections. It can effectively improve the detection accuracy without increasing the detection time.

After the feature extraction, the region proposal network (RPN) is used to extract the ROI in the mask R-CNN. The RPN network outputs rectangular candidate regions that may have defects in the image. The RPN mainly generates multiple anchors on the original image, and then classifies and regresses the generated anchors. The time needed for the RPN network to generate candidate frames is short, significantly reducing the computing resources and achieving real-time detection of wood defects.

Next, RoI Align is applied to derive smaller feature maps from the ROI, which was extracted from feature maps and RPN, and then these smaller feature maps are input to the FC layers. Finally, a softmax classifier and frame regression are used to obtain the type and location of the defect, respectively. RoI Align uses a bilinear interpolation algorithm to determine the feature value of each point in the original image interest area and then performs pooling and other operations. It makes the pixels in the original image, and the pixels in the feature map are entirely aligned, effectively avoiding the quantization operation of ROI pooling.

At the end of the network, mask R-CNN merges a branch that uses the FCN to predict the target mask. The branch distinguishes between the foreground and background by creating a binary mask for each defect, and then uses the FCN to complete instance segmentation, which meets the requirements of the online wood defect detection.

## 3. Results

### 3.1. Determination of Model Parameters and Structure

#### 3.1.1. Glance Network Searched Structure

To construct and test the proposed defect detection model, the 2838 collected wood veneer pictures were divided into three parts including a training set, a testing set, and a validation set in a ratio of 3:1:1. The training set was used to train the constructed network, the testing set was used to evaluate the network, and the validation set was used for the final judgment. The training platform is shown in Table 3.

**Figure 10:**
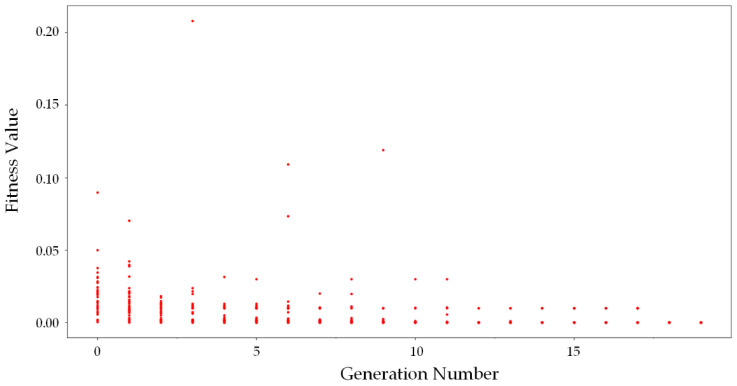
Neural architecture searching process of glance network. The red points represent the networks constructed by the algorithm.

The searching process of the glance network is shown in Figure 10. There are various evaluation values in the initial generation, which shows the variance of the generated network structures. With the growth of the generation number, the network points gather together slowly, although some points are out of the rule due to mutation. In the end, all the networks tended to have similar fitness values, which means that the algorithm reached its convergence. The fitness value converges to a point that represents the fitness value of the glance network.

The search space of the network was massive, and it was difficult to validate whether the result of the searched structure was the best possible. Hence, a comparative experiment was carried out to validate whether the searched result was the local optimization. Table 4 lists the results with different parameters for the glance network. Each row corresponds to a structure of the network, and each column corresponds to one kind of parameter. Model 2 is the glance network determined by NAS, and other models are constructed based on Model 2. By comparing the results of all models, we found that the number of filters strongly affects the accuracy of the model. Fewer filters can effectively decrease the FLOPs but the accuracy also decreases, which is not acceptable for wood defect detection in our study, even though the accuracy is more than 90% [47]. The kernel size also slightly affects the accuracy of the model but it has a strong effect on the FLOPs, as found by comparing Models 4 and 5. The pool size of the max-pooling layer is another central point for model performance, and bigger or smaller pool sizes are not suitable for the application of wood defect detection. The classification accuracy of the glance network plays a decisive role in the performance of the whole network. If the glance network mistakenly classifies a defective image as a regular image, it will cause the defect to be undetectable, which will considerably reduce the accuracy of the entire model. Therefore, the detection rate of the glance network must be as high as possible, even at the cost of more false alarms.

To ensure that the detection rate of the glance network is suitable for as many kinds of applications as possible, an adjustable confidence rate is proposed, expressed as:

(8) $$r=\left\{ \begin{array}{l} 1,y_{1}-y_{2}>\alpha \\ 0,y_{1}-y_{2}\leq \alpha \end{array}\right.$$

where $y_{1}$ and $y_{2}$ are the output of each class, α is the confidence rate chosen by users, and r is the result. A higher confidence rate makes the model more likely to classify the input to class 1, and a negative confidence makes the model tend to classify the input to class 0. Table 5 lists the effect of different confidence rates and their corresponding results. The goal of the confidence rate is to make the detection rate as high as possible. When the confidence rate is set to 0, which is the same as the traditional one, the detection rate is 99.75%. However, in industry usage, the detection rate needs to be higher. A low confidence rate, such as 0.5, seems to have no effect on our dataset. When the confidence rate is 0.9, the false alarm rate increases, and the detection rate reaches 100%. This shows that the model tends to find defects more precisely at the cost of a higher false alarm rate. In the proposed detection method, the high false alarm rate can be solved by the subsequent mask model.

#### 3.1.2. Channel Selection for Multi-Channel Mask R-CNN Input

**Figure 11:**
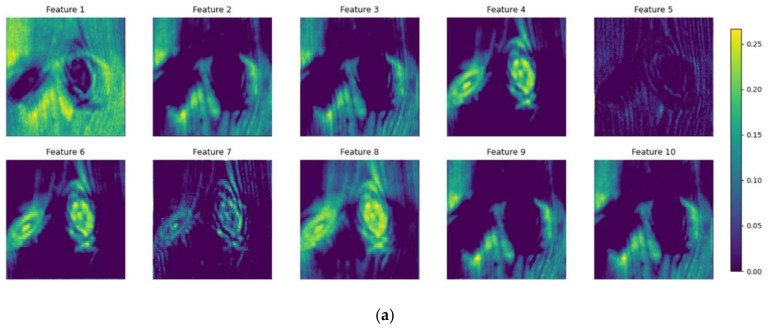

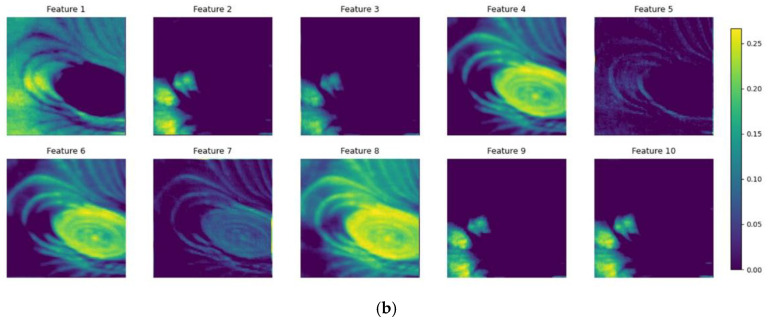
Visualization of the output features of two defect pictures extracted by different convolution kernels. (**a**) Features of an image with two live knots, (**b**) Features of an image with one live knot.

Different kinds of convolution filters extract features from different perspectives, and these features of different channels after Maxpooling2D layer of Glance Network in Figure 9 are visualized together, as shown in Figure 11. Some filters extract the feature from the background, such as Feature 1. Some filters focus mainly about the details of the input pictures, such as Feature 5. There are also some filters that focus on defects, like Features 4 and 8. Some pairs of features are similar to each other, like Features 2 and 3 and Features 9 and 10.

To obtain the best combination of input features for the multi-channel mask R-CNN, the genetic algorithm was used to optimize the selection. Finally, Features 3–5 and 8 were selected and combined with the input feature maps into the multi-channel mask R-CNN. With the selected features, the accuracy of the proposed model reached 98.70%.

An experiment was carried out to determine the influence of multi-channel features on speed performance, and the results are listed in Table 2. The mask R-CNN with different numbers of channels was used to detect five different pictures to obtain the average inference time. The result showed that more channels of feature input to the network slightly increase the average inference time, at most 12 ms, which is an acceptable price for the accurate defect detection.

### 3.2. Classification Performance Evaluation

In this research, the overall classification accuracy (OCA), mean average precision (MAP), and inference time for each batch were used as the evaluation indices of each model. The network classification evaluation indices are:

(9) $$\mathrm{OCA}=\frac{\sum_{i=1}^{k}T_{ii}}{\sum_{i=1}^{k}\left(T_{ii}+B_{i}+\sum_{j=1,j\neq i}^{k}T_{ij}\right)}$$

(10) $$\mathrm{MAP}=\frac{\sum_{i=1}^{k}P_{ii}}{\sum_{i=1}^{k}\sum_{j=1}^{k}P_{ij}}$$

where $P_{ii}$ is the class i pixel predicted to be class i, $P_{\mathrm{ij}}$ is the class i pixel predicted to be class j, $T_{\mathrm{ii}}$ is the class i defect predicted to be class i defect, $B_{i}$ is the class i defect predicted to be the background, and $T_{\mathrm{ij}}$ is the class i defect predicted to be class j defect.

Table 6 presents the inference time for each batch, the overall classification accuracy (OCA), and the mean average precision (MAP) for each model, where the standard deviation of MAP for each model is obtained by calculating the MAPs of all defective images. Notably, each image batch contained 100 images, and 50% of the images had defects, while the others were without detects. The SegNet was the slowest among these models, and thus it is not suitable for online wood defect detection. The FCN was 10.4 s faster than SefNet, but its MAP was 1.6% lower than SegNet. Therefore, the traditional network cannot maintain the inference MAP with a lower cost of inference time. However, the newly designed mask R-CNN has the advantages of both the inference time and the MAP. The proposed method (GM-Mask R-CNN) effectively decreases the inference time of the mask prediction and also improves the MAP of the whole network by introducing the first impression into the following mask network. On the other hand, the higher MAP and the higher MAP standard deviation of the proposed method implies that the proposed method performs better in general, but at the same time is coupled with more extreme outliers (marking defect deviation). In other words, the higher complexity of the detective model may improve detective accuracy, but decrease the stability of model performance, which may be compromised by adjusting training strategies or training parameters.

**Figure 12:**
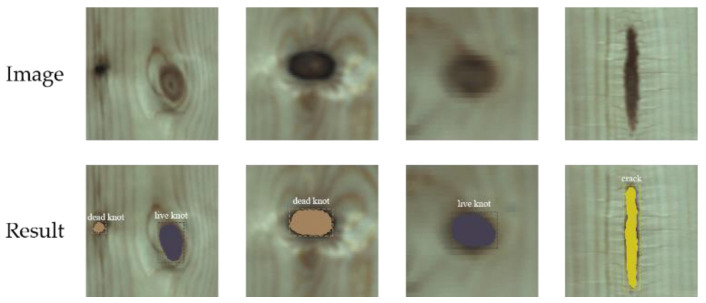
Detect results of the GM-Mask R-CNN.

Figure 12 shows the detective results for each image including the types and locations of the defects. Table 7 lists the OCAs of the three types of defects and background by the proposed method. The classification accuracy of cracks is up to 100% in the testing set, that may because the shape of the crack is distinct from the other two defects. The detective accuracy of live knots is lowest, which failed to be separated as dead knot. Even so, the detective accuracy of live knots is still up to 96.74% in the testing set. Therefore, from these results, we concluded that the detection performance of GM-Mask R-CNN is satisfactory.

## 4. Conclusions and Discussion

A GM-Mask R-CNN model was proposed in this paper for the detection of wood defects, and the experimental results showed that the GM-Mask R-CNN model exhibits excellent performance. The proposed model was used to identify three defects types, including dead knots, live knots, and cracks in the wood; the detection accuracy reached 98.7% and the mean average precision of the model reached 95.31%. Compared with traditional wood defect detection algorithms, the detection accuracy was significantly improved and the running time was reduced. The developed model offers two main improvements as follows:

(1) Improvement of the detection speed of the model: A glance network was designed at the front end of the multi-channel mask R-CNN, which was mainly used to classify regular wood and defective wood. The defective pictures were then picked out and transformed into mask R-CNN for further inspection. To obtain the most suitable architecture of the glance network for wood detection, NAS technology was used to determine the architecture and parameters of the glance network, and FLOPs were used for speed optimization in a NAS for the first time.
(2) Improvement of the detection accuracy of wood defects: We fed the feature of the defective wood extracted by the glance network into the mask R-CNN. In addition, a genetic algorithm was used to optimize the selection of the feature channels to obtain the best combination of input features for the mask R-CNN.

The experiment also provided us with some inspirations for future work. Firstly, the NAS is used to design an appropriate structure for the glance network, and this process will takes much time in the training process, especially when the network searching ranges further increases. We will try to design a more appropriate search strategy to accelerate the search process of glance network structure.

Second, the proposed method is a combined model; the performance of the glance network has great influence on the subsequent classification accuracy of the multi-channel Mask R-CNN, but it is difficult to check which parts diminish the final performance more directly from the finally detective results, because both the glance network and multi-channel Mask R-CNN provide the abstract features to detect defects. Therefore, we will try to find out a joint optimization approach to reduce the whole complexity of the proposed method.

Third, we will continue to try to improve the detection speed of the defect detection algorithm as much as possible and make it capable of detecting more different types of defects. Additionally, the proposed model will be used to calculate the area of the defect, which would lay a good foundation for the classification of wood defects. Algorithms that meet the needs of industrial production must be effectively combined with industrial equipment and put into industrial production.

## Figures and Tables

**Table 1:** Parameters of code blocks for a neural network layer.

Parameter	Range	Type	Parameter	Range	Type
Activation Bit	0: Inactive 1: Active	Enumerated	Strides	1–5	Integer
Layer Type	1: Cov2D + Maxpooling 2: Dropout	Enumerated	Pool Size	2–8	Integer
Filter Number	1–128	Integer	Pool Strides	2–6	Integer
Kernel Size	1–8	Integer	rate	0–1	Float

**Table 2:** Comparison results for different numbers of features.

Feature Number	Load Time(s)	Picture 1 (s)	Picture 2 (s)	Picture 3 (s)	Picture 4 (s)	Picture 5 (s)	Average Inference Time(s)
0	1.169	0.039	0.035	0.037	0.048	0.041	0.040
1	1.153	0.049	0.038	0.037	0.040	0.046	0.042
2	1.143	0.043	0.039	0.044	0.050	0.043	0.043
3	1.140	0.042	0.042	0.040	0.044	0.046	0.044
4	1.178	0.043	0.044	0.043	0.046	0.053	0.046
5	1.140	0.049	0.046	0.043	0.046	0.046	0.046
6	1.148	0.057	0.048	0.045	0.049	0.047	0.047
7	1.137	0.049	0.047	0.046	0.047	0.048	0.049
8	1.135	0.051	0.053	0.049	0.052	0.048	0.050
9	1.176	0.050	0.050	0.048	0.051	0.050	0.051
10	1.169	0.052	0.051	0.051	0.052	0.056	0.052

**Table 3:** Hardware and software parameters of the experimental environment.

Name	Parameter
Memory	32.00 GB
CPU	Intel Core i7-8700 CPU @ 3.2 GHz
Graphics card	NVIDIA GeForce RTX 2080 Ti
System	Linux Ubuntu 18.04 LST
Environment Configuration	Python3.6, TensorFlow-GPU 1.14.0, Keras2.0.8

**Table 4:** Comparison results for different glance network structures.

Model Order	Number of Filters	Cov2D Kernel Size	Maxpooling2D Pool Size	Detection Rate	False Alarm	Model Accuracy	FLOPs(M)
1	6	3	2	99.51%	5.9%	96.76%	14.381
2	10	3	2	99.75%	0%	99.88%	23.968
3	14	3	2	99.51%	9.2%	95.14%	33.555
4	10	2	2	99.50%	0.76%	99.37%	11.984
5	10	5	2	99.75%	7.8%	95.94%	62.336
6	10	3	1	99.75%	5.7%	97.01%	22.792
7	10	3	4	99.75%	7.6%	96.03%	28.673

**Table 5:** Comparison results for different confidence rates.

Confidence Rate	Detection Rate	False Alarm	Model Accuracy
0	99.75%	0.87%	99.44%
0.5	99.75%	0.87%	99.44%
0.9	100%	2.6%	98.7%

**Table 6:** Comparison of detection results for different networks.

Method	OCA (%) ^1^	MAP (%) ^2^	Inference Time/Batch (s)
GM-Mask R-CNN (Resnet50) ^3^	98.70	95.31 ± 4.5	2.5
Mask R-CNN (Resnet101)	98.52	93.32 ± 3.2	6.1
SegNet [48]	98.45	92.27 ± 3.9	20.1
FCN ^4^ [49]	98.45	90.67 ± 4.1	9.7

^1^ OCA is overall classification accuracy. ^2^ MAP is mean average precision. ^3^ GM-Mask R-CNN is glance network and multiple channels mask region convolution neural network. ^4^ FCN is fully convolutional neural network.

**Table 7:** Confusion matrix of defect type OCA detected by the proposed method.

Defect Types	Live Knot	Crack	Dead Knot	Background
Live knot	96.74%	0.93%	2.33%	0.00%
Crack	0.00%	100%	0.00%	0.00%
Dead knot	0.87%	0.00%	99.13%	0.00%
Background	0.00%	0.00%	0.99%	99.01%
